# Mechanisms of hypoxia in the hippocampal CA3 region in postoperative cognitive dysfunction after cardiopulmonary bypass

**DOI:** 10.1186/s13019-022-01865-z

**Published:** 2022-05-07

**Authors:** Ting Liu, Rui Deng, Xin Wang, Ping Liu, Qiu-Xia Xiao, Qing Liu, Ying Zhang

**Affiliations:** 1 Department of Anesthesiology, Affiliated Traditional Chinese Medicine Hospital, Southwest Medical University, Luzhou, 646000 China; 2 Laboratory of Anesthesiology, The Affiliated Hospital of Southwest Medical University, Luzhou, 646000 China; 3 Department of Anesthesiology, People’s Hospital of Deyang City, Deyang, 618000 China

**Keywords:** Cardiopulmonary bypass, Postoperative cognitive dysfunction, Hypoxia, Blood brain barrier, Central nervous system

## Abstract

**Background:**

Postoperative cognitive dysfunction (POCD) is a complication with high morbidity and mortality, commonly observed in the elderly who underwent anesthesia and surgery. The incidence is much higher in cardiac surgery. However, the reason and the mechanism of POCD remains unclear, but cerebral hypoxia is a common neurological complication after cardiac surgery. This study aims to investigate what role cerebral hypoxia plays in the pathogenesis of POCD.

**Methods:**

The POCD model was established using cardiopulmonary bypass (CPB) surgery. Cognitive function was detected using Y maze and Morris water maze. The hypoxia in central nervous system was assessed using HE staining, western blot, and immunofluorescence. Inflammatory factors in hippocampus and plasma were detected by enzyme-linked immunosorbent assay. Evans blue was used to detect destruction of the blood brain barrier (BBB).

**Results:**

Cognitive impairment markedly occurred to rats underwent 2-h CPB operation. Cerebral thrombosis and hypoxia occurred in the hippocampal CA3 region of rats after surgery. In addition, microglia in hippocampal was activated and the expression of inflammatory factors such as IL-1β, IL-6 and TNF-α was upregulated. Moreover, the permeability of BBB increased in rats after CPB.

**Conclusion:**

Hypoxia in hippocampal CA3 region was involved in the occurrence and the mechanism may be associated with neuroinflammation and the damage of BBB.

## Introduction

Postoperative cognitive dysfunction (POCD) as a common central nervous complication after surgery, refers to the central nervous system complications after surgery, manifesting as mental disorder, anxiety, personality changes and memory impairment [1]. The risk factors of POCD commonly include age [2, 3], level of education [4], types of anesthesia and surgery, etc. The incidence of POCD is as high as 25.8% 1 week and 9.9% 3 months after noncardiac surgery [5], while it is significantly higher as 59% after cardiac surgery [6]. Though many scholars have been studying the mechanism of POCD for long time, it still remains unclear yet. Similarly, the reason for the higher incidence of POCD after cardiac surgery is also not really clear. Previous studies have shown that cerebral infarction is involved in the pathogenesis of cognitive decline [7, 8].

Cerebral infarction is a common neurological complication after cardiac surgery [9], The incidence of recessive cerebral infarction detected by diffusion-weighted magnetic resonance imaging is high as 50% [10]. Microemboli from cardiac surgery can block blood flow and cause ischemia and capillary loss [11], and hypoxia. As we all know, hypoxia is commonly companied with inflammation and cell apoptosis. Moreover, neuroinflammation has been demonstrated to be involved in the pathology of POCD [12, 13]. Nevertheless, it needs more and deeper investigation that whether cerebral infarction happens in the hippocampus or in other regions and how cognitive function will change with hypoxia in hippocampus.

In the present study, postoperative cognitive function was detected after CPB surgery and the relationship between cerebral hypoxia and POCD was investigated.

We present the following article in accordance with the ARRIVE Checklist.

## Materials and methods

### Animals

All animal experiments were approved by the animal ethics committee of Southwest Medical University and performed in accordance with the National Institute of Health guidelines and regulations. Adult male SD rats (n = 105) weighing 250–300 g were purchased from Chengdu Dossy Experimental Animals Co., Ltd. (License Number: SCXK (CHUAN) 2015-030). All of them were healthy without mental retardation, housed in Laboratory animal center of Southwest Medical University under controlled laboratory conditions and given free access to sufficient food and water. The air-conditioned room was set at 20–25 °C with 45–60% humidity, under a standard 12–12 light–dark cycle (lights on 7 am. to 7 pm.).

Experiment consisted of two parts. For the first part, fifteen healthy male rats were selected and divided randomly and equally to the cardiopulmonary bypass for an hour (CPB 1 h) group, the cardiopulmonary bypass for 2 h (CPB 2 h) and the sham operation (SHAM) group, with five rats in each group to assess the better surgery duration for POCD model. For the second part, ninety healthy male SD rats were randomly divided into the 2 h CPB group and the Sham group, with forty-five rats in each group to assess change after anesthesia and surgery.

### Animal model of POCD

The POCD model of rats was established using CPB surgery described previously [14], with minor modifications. Briefly, after being anesthetized with 30 mg/kg pentobarbital through an intraperitoneal injection, rats were inserted into a tracheal tube, which was connected to the ventilator. After a longitudinal incision was made in the middle of the neck, the right external jugular vein was separated and the catheter using an indwelling needle was inserted into the right atrium. The left common carotid artery was also separated, and an indwelling needle as a catheter was inserted into the skull. Then the peristaltic pump was started and pre-infusion solution was added into the extracorporeal circulation pipeline (membrane lung + pipeline) to exhaust the air in the pipeline. 4 ml pre-charge liquid was composed of ringer lactate solution: normal saline: hydroxyethyl starch:Mannitol:Sodium bicarbonate in a 11:7:1:1 ratio. 400 IU/kg heparin was injected through the right external jugular vein, and animal membrane lungs was connected for pure oxygen. After that, extracorporeal circulation was started. During the operation, the peristaltic pump was adjusted to maintain the average flow rate between 10 and 12 mL/kg/min, and the average arterial pressure of the rat was maintained at 60–100 mmHg. A heating blanket was used to keep the rat body temperature at 37–38 °C. It stopped after 120 min of commuting. Before the end of CPB, 0.5 mg protamine injection, 0.2 mg furosemide injection and 0.8 mg gentamicin injection were injected through the circulation pipeline. Then the tubes were pulled out and the skin was sutured. The rats from the Sham group were only after anesthetized, the common carotid artery and the right atrium were isolated and ligated, not subjected to CPB surgery.

### Morris water maze (MWM) test

Morris water maze test was used to investigate spatial learning and memory of rats [15], which consisted of positioning navigation test and space exploration test. The device consisted of a swimming pool with a depth of 60 cm and a diameter of 200 cm. Inside, clean cold water and hot water were mixed to maintain water temperature at about 23 °C, and water depth was kept at about 40 cm. A hidden platform with a diameter of 21 cm was located in one of the quadrants and about 1 cm below the water surface. During the whole test, the hidden platform’s position stayed unchanged. In the experiment, each rat was placed in a quadrant in the water, swimming freely in the pool until it found and climbed up the platform. The time from rats being placed in the water to finding the platform and swimming paths were recorded automatically by a camera system. Then, they were analyzed by a corresponding software. If the rat didn't find the platform in 90 s, it would be guided to climb up the platform and remained for 30 s to remember the markers surrounded. All rats accepted the test 4 times a day for 5 consecutive days after 24 h of CPB. Then the sixth day after CPB, the platform was removed and each rat was allowed to swim for 120 s. The time between rats being placed in the water and finding the target, as well as the platform-crossing times was recorded.

### Y maze

Y maze test was also used to assess spatial memory [16]. The Y maze consisted of 3 equal arms (50 cm × 18 cm × 35 cm), with an angle of 120° between each arm. The inner arms and the bottom of the maze were painted black and a movable partition was placed at the center of each arm. In the experiment, rats were placed at the end of either arm and allowed to explore freely for 8 min. The shuttle path of rats was recorded by a camera. After each test, the feces and urine discharged by the rats in the Y maze were cleaned to avoid the influence on the test results. The total number of entries, an alteration and the number of maximum alternations were recorded. Spontaneous alteration was calculated by a corresponding software.

### Hematoxylin and eosin (HE) staining

HE staining was used to investigate thrombosis in hippocampus. 4 μm longitudinal sections of hippocampal tissues were dehydrated with alcohol, and added with water. Then, the sections were stained with hematoxylin solution for 6–8 min, rinsed in the reverse side with running water, stained with eosin for 10 s, and then rinsed again. Afterward, the sections were dehydrated with graded alcohol and cleared in xylene. A light microscope was used to observe pathological changes of the hippocampal tissues.

### Immunofluorescence

Immunofluorescence was used to detect the content of hypoxyprobes in hippocampus. The rats were anesthetized by isoflurane and sacrificed, and were perfused with PBS through the heart. Rat brains with the hippocampus were fixed with 4% paraformaldehyde (Beijing Solarbio Science & Technology) and dehydrated with 30% sucrose solution. Optimal cutting temperature (OCT) compound (Sakura, USA) was used to wrap the rat brain tissue and slices of 20 μm were cut along the coronal plane. The slices were immersed in phosphate buffer saline (PBS) containing 10% goat serum (Beijing Solarbio Science & Technology) and 0.3% triton-100 at 37 °C for 30 min and then, the primary antibody (Iba-1, 1:500, Abcam, UK) was added to incubate the sections at 4 °C for 16 h. The following day, after the slices were washed in PBS, the secondary antibody (Goat anti-rabbit, 1:1000, Abcam, UK; Goat anti-mouse, 1:1000, Abcam, UK) was used to incubate the sections at room temperature for 2 h in a dark room. Then, the slices were washed again in PBS and soaked with 4′,6-diamidino-2-phenylindole (DAPI) Beijing Solarbio Science & Technology) for 15 min. Then, the slices were washed with PBS, added with antifade mounting medium, and observed slices of brain tissue Using Olympus microscope BX50 and acquired images.

### Western blot

Western blot was used to detect the amount of hypoxyprobe-1 in the hippocampus. Radio-immunoprecipitation assay lysis buffer (RIPA, Beijing Solarbio Science & Technology) containing 2 ug/ml protease inhibitor (Beijing Solarbio Science & Technology) was added to the hippocampus tissue of rats and tissue was grinded to make a homogenate on ice, which was centrifuged next. After collection of the supernatant, protein was quantified using the Bicinchoninic acid (BCA) reagent (Beyotime, Shanghai, China). Then, the proteins were separated by 10% SDS-PAGE for electrophoresis and transferred onto a polyvinylidene fluoride (PVDF) membrane (Millipore, America). After washed in 0.1% Tris–HCl buffer solution and Tween (TBST), the membranes were blocked with 5% skim milk at room temperature for 1 h. Next, the membranes were incubated by anti-hypoxia probe—1 antibody (1:100, hypoxia probe, America) at 4° C under wave incubation bed overnight. The following day, the membranes were washed with 15 mL TBST three times for 5 min each time and was incubated with secondary antibody diluent (1:500, Absin, China) with 10 mL blocking buffer by gently shaking at room temperature for 1 h. Then, the membranes were washed again with TBST and the ECL luminescent solution (Beyotime, Shanghai, China) was added to the membranes to incubate for 1 min. Afterward, the membranes were expose to X-ray film and the pictures were captured by a corresponding system.

### Enzyme-linked immunosorbent assay (ELISA)

Elisa was used to detect inflammation after surgery. The same weighted hippocampus tissue of rats was grinded to make a homogenate on ice and the sample supernatant was obtained. The levels of interleukin-1beta (IL-1β), interleukin-6 (IL-6), tumor necrosis factor-alpha (TNF-α0 in hippocampal and plasma were detected using the ELISA-kit (Qiaoshe, Shanghai, China) according to the manufacturer’s instructions.

### Evans blue staining

Evans blue was used to detect the damage of BBB. 1 mL 2% Evans blue was injected through a tail vein 2 h before the rats were put to death. After anesthesia, the rats were sacrificed, the hearts were perfused with normal saline until the flowing liquid became clear and transparent, then the brain was cut off. The brain was evenly cut into 5 slices along the coronal plane, and the brain was observed and photographed. The brain tissue was put into a centrifuge tube and 1 mL PBS was added. The tissue was rapidly homogenized with a tissue homogenizer and centrifuged and the supernatant was taken. The supernatant was added with same amount of trichloroacetic acid and incubated at 4 °C for 24 h. After being centrifuged for 15 min, the supernatant was taken. Then the absorbance at 620 nm was measured with an enzyme marker, and the absorbance value of Evans blue solution with different concentration standard was determined and the standard curve was drawn at the same time. The concentration of Evans blue in each sample was calculated according to the standard curve formula.

### Statistical analysis

SPSS 20.0 statistical software was used to analyze the data. The measurement data is expressed by the mean ± standard deviation (SD), and the effect analysis of grouping and time adopts two-factor analysis of variance. If there is an interaction effect, t-test is used for analysis between groups, one-way ANOVA is used for multiple time point analysis, and the LSD method is used for pairwise comparison of time points. *P* < 0.05 is considered statistically significant.

## Results

### CPB for 2 h induced obvious cognitive impairment

**Fig. 1:**
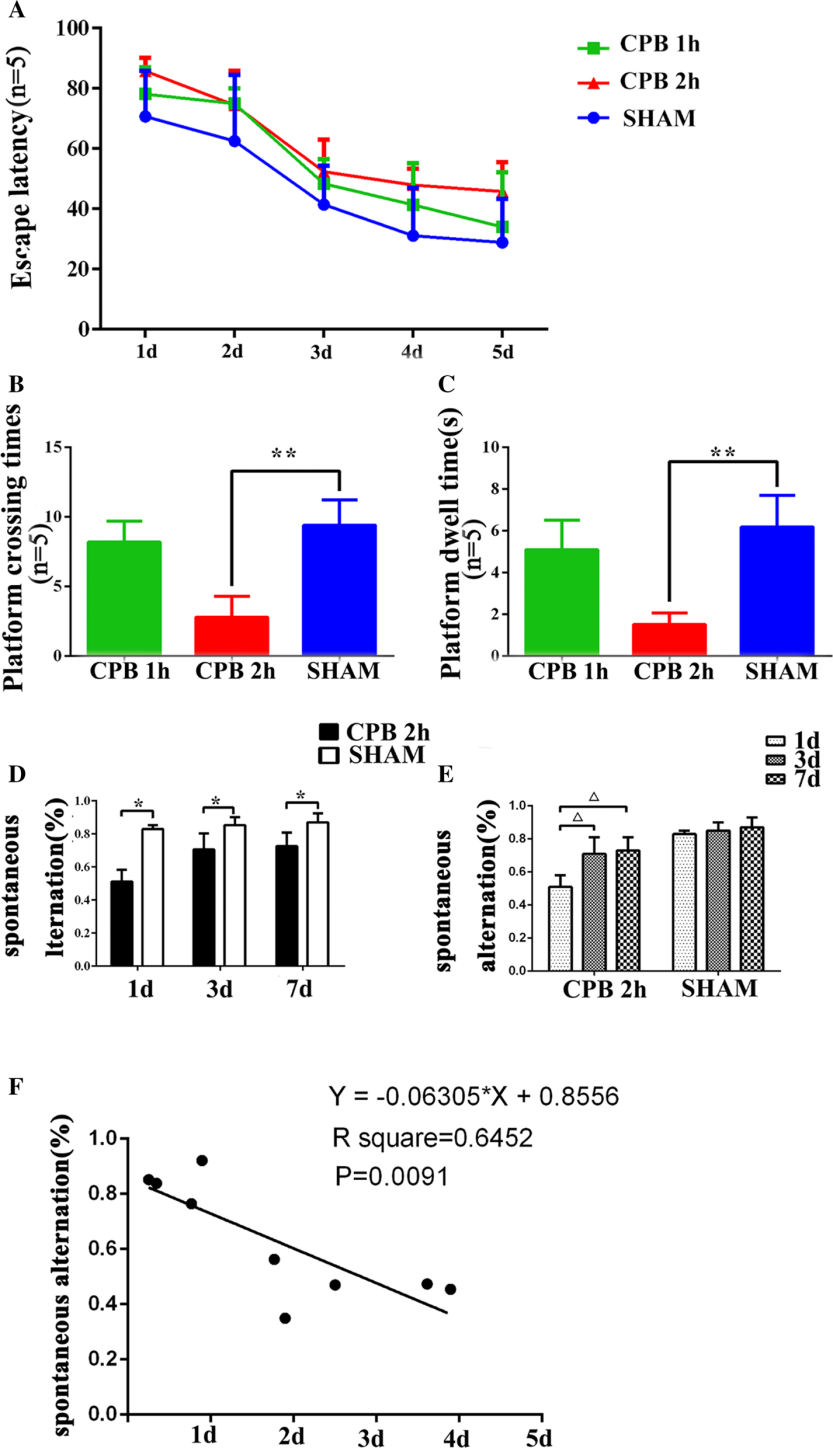
Cognitive function test after surgery. The results of the water maze experiment in the CPB group and the SHAM group after the operation including escape latency (**a**) and platform crossing times (**b**) and platform dwell times (**c**); The Y maze spontaneous alternations test examined the working memory of the two groups of rats (**d**, **e**). Correlation analysis between Y Maze spontaneous alternations and the content of hippocampal hypoxia in rats (**f**). **P < 0.05, *P < 0.01 compared with the SHAM group; Δ*P* < 0.05 compared with the 1 d. *CPB* cardiopulmonary bypass, *h* hour, *d* day, *s* seconds

Morris water maze were carried out on rats after surgical modeling. It was found that rats in CPB 2 h and CPB 1 h had a little longer escape latency (Fig. 1a) compared to the SHAM group. The CPB 2 h group had more platform crossing times (Fig. 1b, *P* < 0.05) and less platform dwell time (Fig. 1c, *P* < 0.05) compared to the SHAM group. However, there was no significant difference between the CPB 1 h group and the SHAM group (Fig. 1b, c, *P* > 0.05). Y maze was also used to detect cognitive changes of the rats. In the experiment, the rats in the CPB 2 h group performed significantly worse (less spontaneous alternation, *P* = 0.0091) than rats of the SHAM group (Fig. 1d–f, *P* < 0.05).

### Cerebral embolism and hypoxia happened after CPB in the hippocampal CA3 region

**Fig. 2:**
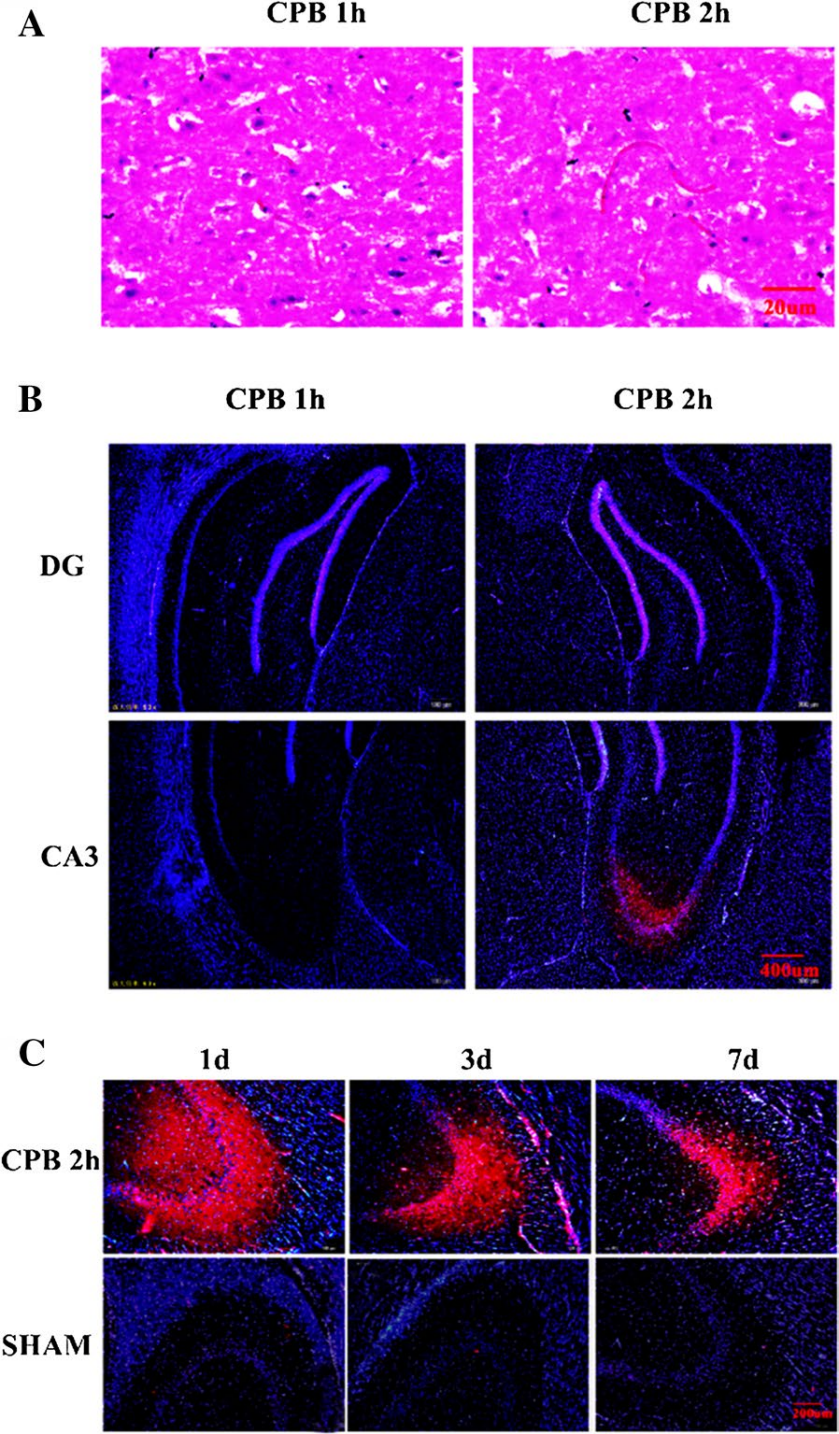
Microthrombus in cerebral vessels of rats. HE staining on the seventh day (**a**), Scale bar = 20 μm; Immunofluorescence staining results of hypoxia tissue in hippocampus CA3 district in CPB group (**b**), Scale bar = 400 μm, DAPI counterstaining (blue) shows nuclei of intact cells, and red fluorescence represented hypoxyprobe-1+; Immunofluorescence staining results of hypoxia tissue in hippocampus CA3 district in CPB group and SHAM group (**c**), Scale bar = 200 μm, DAPI counterstaining (blue) shows nuclei of intact cells, and red fluorescence represented hypoxyprobe-1+. *CPB* cardiopulmonary bypass, *h* hour, *d* day

HE staining on the seventh day after the operation revealed thrombosis in the brain tissue after CPB and cerebral embolism CPB for 2 h caused was more obvious and severe compared to CPB 1 h group (Fig. 2a). Immunofluorescent hypoxia probe was used to detect the hypoxia in the hippocampus. Among the picture, hypoxyprobe-1 was detected in hippocampal CA3 region of the rats in CPB 2 h group, while not appear in the DG region (Fig. 2b). Similarly, hypoxyprobe-1 also didn’t show up in hippocampal DG region and CA3 region of the rats in CPB 1 h group (Fig. 2b). Furthermore, hypoxyprobe-1 was all detected in hippocampal CA3 region of the rats after CPB 2 h on postoperative day 1, 3, and 7, which was gradually decreased from 1 to 7 days and was most obvious in the 1 day in CPB 2 h group. But it was not found in the SHAM group (Fig. 2c).

**Fig. 3:**
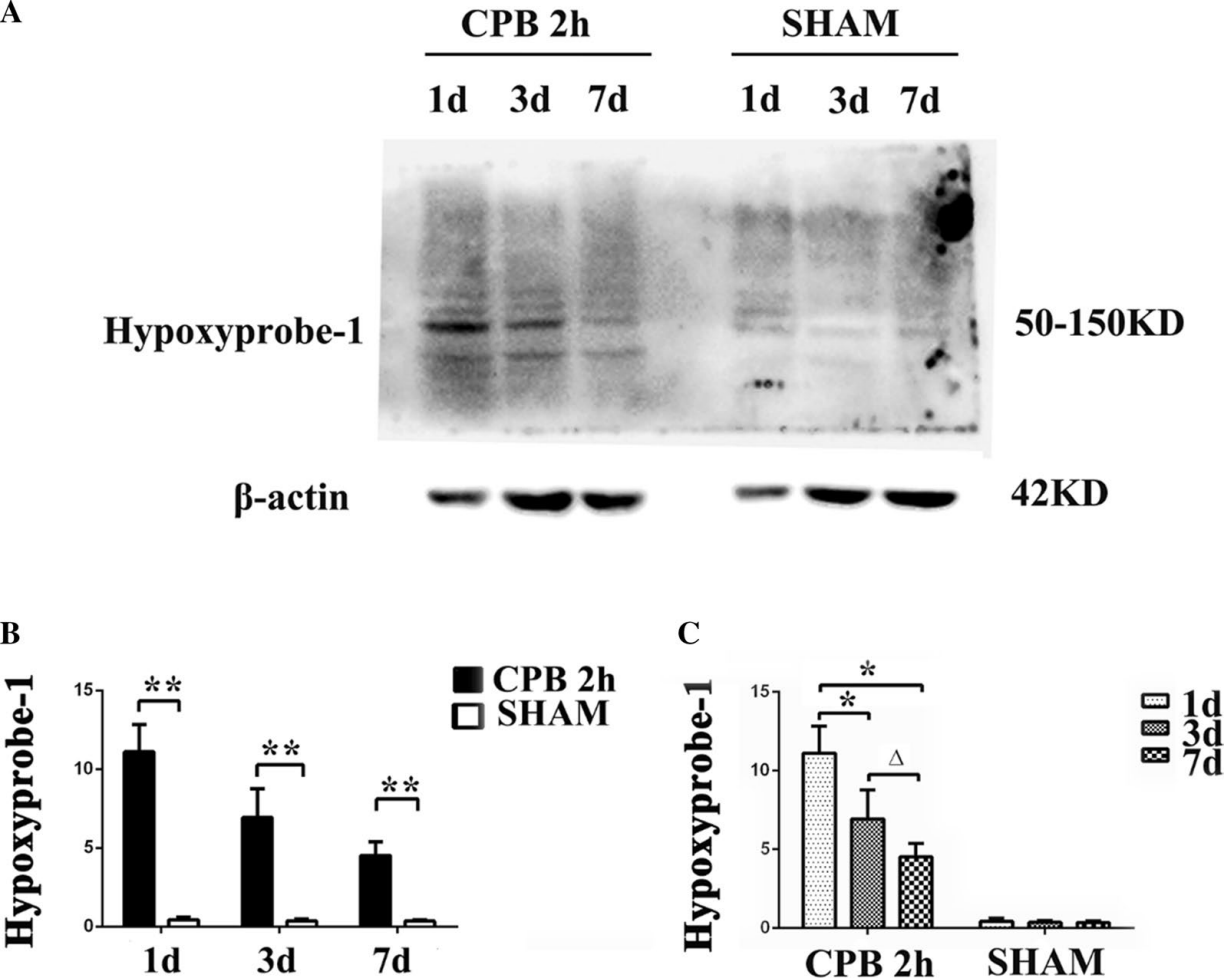
The change of hippocampal anoxia after sugery. Comparison of the content of hippocampal anoxia between the SHAM group and the CPB group at three time points: the first day, the third day, and the seventh day after surgery (**a**–**c**). **P < 0.05, *P < 0.01 compared with the SHAM group; Δ*P* < 0.05 compared with the 3 d. *CPB* cardiopulmonary bypass, *h* hour, *d* day

Western blot was used to detect the amount of hypoxyprobe-1 in the hippocampus. It showed that the content of hypoxia probes in the hippocampus of the CPB 2 h group were significantly higher than that of the SHAM group, and the content of hypoxia probes decreased successively at three time points on postoperative day 1, 3, 7 after surgery (Fig. 3a). The content of Hypoxyprobe-1 was significantly higher in CPB 2 h group than that in the SHAM group on postoperative day 1, 3, 7 (Fig. 3b, *P* < 0.05). In CPB 2 h group, the content of hypoxyprobe-1 was decreased in chronological order at three time point on postoperative day 1, 3, 7, while it didn’t show up in the SHAM group (Fig. 3c, *P* < 0.05).

### Surgical trauma enhanced microglial activation and neuroinflammation

**Fig. 4:**
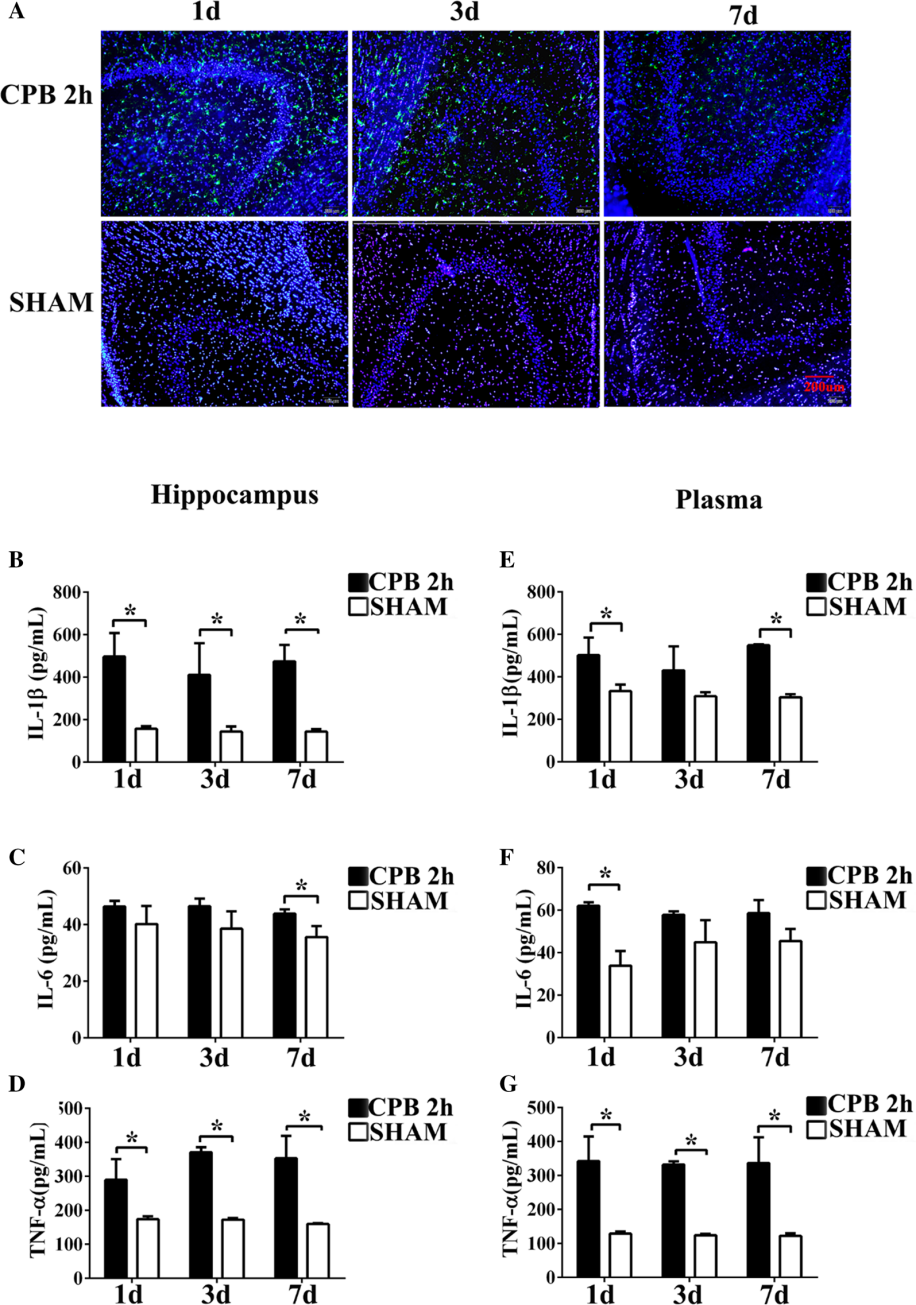
Cardiopulmonary bypass induced inflammation in rats. Immunofluorescence results were obtained on the first day, the third day and the seventh day after the operation of the two groups of rats, green fluorescence indicates Iba-1 (**a**), Scale bar = 200 μm, DAPI counterstaining (blue) shows nuclei of intact cells, and green fluorescence represented Iba-1+. Bar graphs indicate the changes in the levels of IL-1, IL-6, and TNF-α in the hippocampus (**b**–**d**). Bar graphs indicate the changes in the levels of IL-1, IL-6, and TNF-α in the plasma (**e**–**g**). *P < 0.01 compared with the SHAM and the CPB groups. *CPB* cardiopulmonary bypass; *h* hour, *d* day

Immunofluorescence staining (Iba-1) showed the activated proliferation of microglia in the CPB 2 h group on the day 1, 3, and 7 after operation, which was different from the results in the SHAM group. The expression of Iba-1 was significantly increased in the CPB 2 h group on the day 1, 3, and 7 after operation compared with SHAM group (Fig. 4a). The levels of IL-1β, IL-6 and TNF-α in hippocampus of rats were detected by ELISA. Both in hippocampus and plasma, the levels of IL-1β were higher than those in the SHAM group at three time point, and it was more obvious in hippocampus (Fig. 4b, e, *P* < 0.05). The levels of IL-6 were significant different only on the seventh day after surgery in hippocampus and on the first day after surgery in plasma, while in other time points, the difference was not significant (Fig. 4c, f, *P* < 0.05). The levels of TNF-α in CPB 2 h group are significantly higher than those in the SHAM group all on the first day, third day, and the seventh day (Fig. 4d, g, *P* < 0.05).

### BBB has been damaged after CPB surgery

**Fig. 5:**
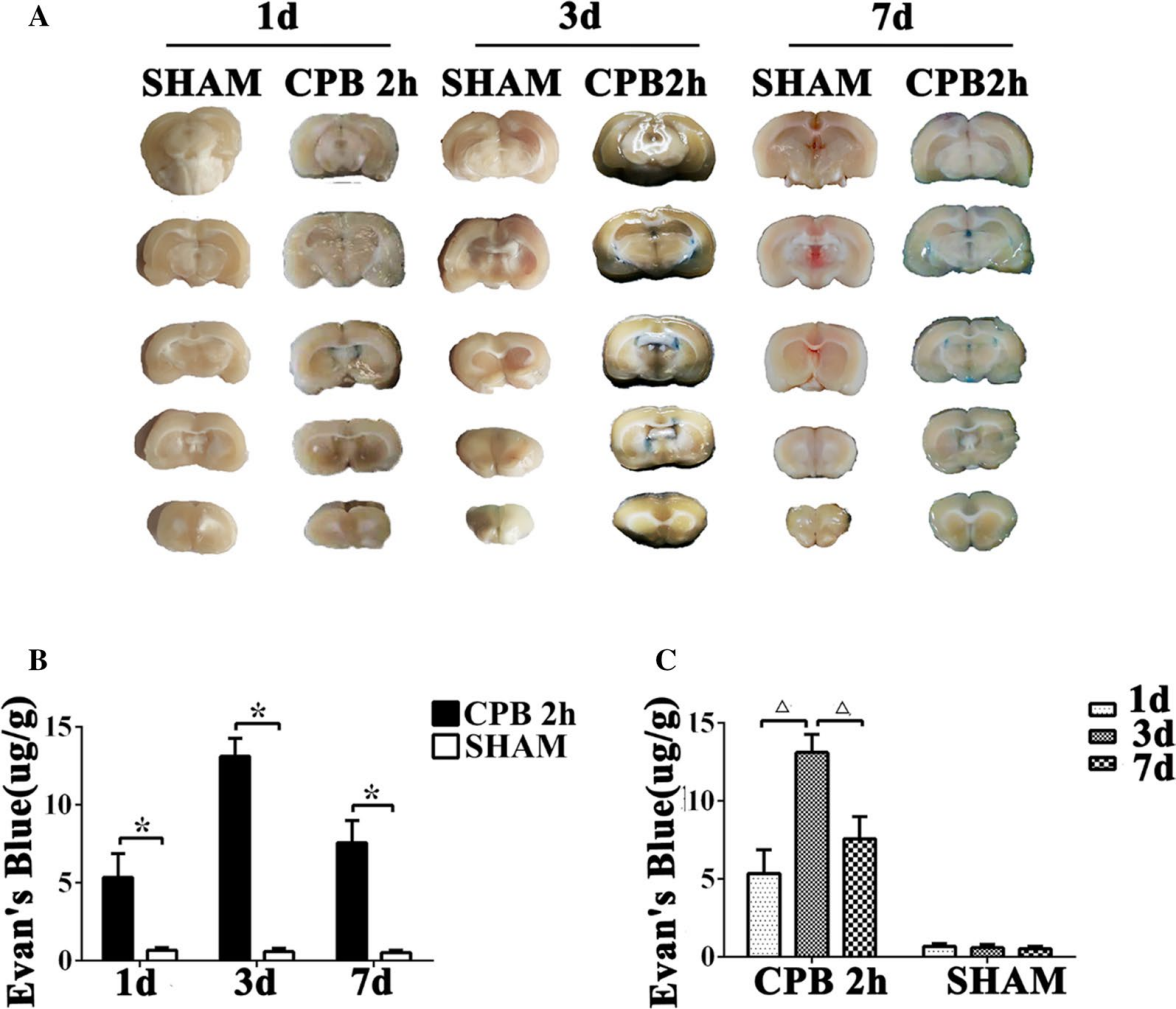
The permeability of blood–brain barrier increased in rats after CPB. Brain tissue is dyed blue by Evans blue, which crosses the blood–brain barrier (**a**). Bar graphs indicate the amount of Evans blue in brain tissue compared between the two groups (**b**). Bar graphs indicate the changes of Evans blue in brain tissue on the first day, the third day, and the seventh day after surgery (**c**). *P < 0.05 compared with SHAM group. *CPB* cardiopulmonary bypass; *h* hour, *d* day

Evans blue staining showed that part of the hippocampal brain tissue of rats in the CPB 2 h group was stained blue. And it was more obvious in the third day and seventh day (Fig. 5a). However, the hippocampus was not stained blue in SHAM group (Fig. 5a). On postoperative day 1, 3, 7, the content of Evans blue in brain tissue of rats was significantly higher in the CPB 2 h group than that in the SHAM group (Fig. 5b, *P* < 0.05). In addition, the level of Evans blue was highest on the third day postoperatively compared to that on the first day and the seventh day after surgery, while there was no significant change of the content of Evans blue in SHAM group at three time point (Fig. 5c, *P* < 0.05).

## Discussion

Many studies showed various duration of CPB surgery used for the model of POCD. Study proved 2-h CPB could lead to inflammatory response in rats [17], while other studies [18, 19] found that approximate 1-h CPB could lead to postoperative cognitive dysfunction in rats. Therefore, we designed the first part of the experiments to detect which model would be better to perform next part of experiments and finally we found out 2-h CPB may be better. This result is the same with Dieleman et al. [20], but different with Mackensen et al. [19]. The difference may show because of age, since age has been confirmed to be one risk of POCD [3]. Besides, lack of sensitivity of the above two behavioral experiments to the detection of POCD, or the low sample size of this experiment may be other reasons. In addition, our CPB model was built with reference to Zhu et al. [14], a model without blood priming, which may be one possible reason. Based on the result, duration of surgery might be a possible influencing factor of the occurrence of POCD but needs more study to provide strong evidence.

Cerebral microembolism after CPB is an important etiologic factor of POCD [21]. Microembolic event rates may be a predictor of cerebral infarction progression [22], indicating that it can lead to cognitive impairment. Our study has found out that hypoxia caused by cerebral microembolism occurred after CPB. In addition, inflammation responses and BBB damaged. According to the results of the experiment, the cerebral hypoxia located in the hippocampal CA3 region. Hypoxia can affect the production of energy [23, 24], can produce a lot of free radicals [23], can degenerate the hippocampus [25], and cause neuronal dysfunction [24]. The hippocampus is the main structure responsible for learning and memory, and hippocampal CA3 output is crucial for consolidation of memory [26]. Arterial vascularization of the hippocampus is dependent on the collateral branches of the posterior cerebral artery and the anterior choroidal artery, while hippocampus CA3 region only accept blood supply from the large dorsal hippocampal artery [27], so the arterial thromboembolism or even their upstream arterial thromboembolism can lead to hypoxia in the CA3 area in the end.

Current studies suggest that the occurrence of postoperative cognitive dysfunction is related to the inflammation of the nervous system [12, 28], the destruction of the BBB [13, 29]. Our study showed that rats with POCD had a central nerve inflammation and microglial cells as the inflammatory cells in central nervous system were activated, which are the same with previous studies [30, 31]. The activation of microglia produces more inflammatory factors, increases damage process, and forms vicious cycle [32]. Microglia are rapidly activated when brain tissue is hypoxic and ischemic, and the activated glia produce a large amount of inflammatory cytokines, including IL-1β, IL-6, TNF-α and nitric oxide [33]. These inflammatory factors are associated with memory impairment [34–36]. They promote inflammation, leading to poor outcomes after ischemic brain injury.

The destruction of the BBB is also a possible mechanism for POCD. The BBB is formed by endothelial cells, astrocytic end-feet processes, perivascular neurons and pericytes [37]. As the initial trigger of pathophysiological changes in the BBB, hypoxia can cause peripheral immune cell infiltration and blood leakage of proteins into the brain. Besides, hypoxia stimulates the structure of blood vessels because it stimulates the proliferation of endothelial cells (ECs), which leads to the formation of new blood vessels and further promotes the activation and proliferation of astrocytes [38, 39]. The destruction of the BBB may result from the activated Matrix metalloproteinase-9 (MM9), which will damage the basement membrane of the capillary vessels through degrading of junctional complexes (Claudin4, Claudin5, VE-Cadherin, and ZO-1) [40]. Moreover, TNF-α can directly destroying BBB by leading to necroptosis of EC [41]. In this study, the permeability of BBB reached the highest on the third day rather than the first day after surgery. This phenomenon may be because the level of TNF-α on the third day after surgery was higher than that on the first day after surgery, further aggravating the damage of the BBB. However, it may also be because of biphasic opening [42]. CPB triggers cerebral ischemia and hypoxia and inflammation, increasing release of inflammatory factors such as IL-1β, IL-6, TNF-α [43]. And these inflammatory factors can induce leukocytes to cross the damaged BBB and enter the brain tissue (44).

## Conclusion

Our study shows that cardiac surgery such as CPB leads to a large amount of microembolism, which can cause hypoxia in the CA3 region of the hippocampus. Hypoxia in hippocampal CA3 region develops POCD by inducing the inflammation and damage the BBB.

## Data Availability

The datasets used and/or analyzed during the current study are available on request.

## Abbreviations

POCD: Postoperative cognitive dysfunction
CPB: Cardiopulmonary bypass
CNS: Central nervous system
BBB: Blood brain barrier
MWM: Morris water maze
HE: Hematoxylin and eosin
OCT: Optimal cutting temperature
DAPI: 4',6-Diamidino-2-phenylindol
RIPA: Radio-immunoprecipitation assay lysis buffer
PBS: Phosphate buffer saline
TBST: Tris–HCl buffer solution and Tween
PVDF: Polyvinylidene fluoride
ELISA: Immunosorbent assay
IL-1β: Interleukin-1beta
IL-6: Interleukin-6
TNF-α: Tumor necrosis factor-alpha
ECs: Endothelial cells
MMP9: Matrix metalloproteinase-9
SD: Standard deviation
EC: Endothelial cell
